# Unite to divide: Oligomerization of tubulin and actin homologs regulates initiation of bacterial cell division

**DOI:** 10.12688/f1000research.13504.1

**Published:** 2018-02-28

**Authors:** Marcin Krupka, William Margolin

**Affiliations:** 1 Department of Microbiology and Molecular Genetics, McGovern Medical School, Houston, USA

**Keywords:** cytokinesis, bacteria, Escherichia coli, FtsZ, FtsA, protofilaments

## Abstract

To generate two cells from one, bacteria such as *Escherichia coli* use a complex of membrane-embedded proteins called the divisome that synthesize the division septum. The initial stage of cytokinesis requires a tubulin homolog, FtsZ, which forms polymers that treadmill around the cell circumference. The attachment of these polymers to the cytoplasmic membrane requires an actin homolog, FtsA, which also forms dynamic polymers that directly bind to FtsZ. Recent evidence indicates that FtsA and FtsZ regulate each other’s oligomeric state in *E. coli* to control the progression of cytokinesis, including the recruitment of septum synthesis proteins. In this review, we focus on recent advances in our understanding of protein-protein association between FtsZ and FtsA in the initial stages of divisome function, mainly in the well-characterized *E. coli* system.

## Introduction

For a cell even as simple as *Escherichia coli*, splitting in half is a complex maneuver that needs to be finely regulated in time and space. For this purpose, *E. coli* has evolved a protein complex (the divisome) that spans the cytoplasmic membrane and forms a circumferential ring around midcell ^1^. Seen in cross-section, the innermost part of this complex, termed the proto-ring, consists of membrane-anchored cytoskeletal proteins that form polymers on the cytoplasmic side ^2^. These polymers interact with other proteins that span the membrane and guide the inward synthesis of cell wall material, called the division septum, which eventually will close to form the poles of the newborn cells. Within the proto-ring, FtsZ forms a dynamic scaffold for the recruitment of other septum synthesis proteins. FtsA and another protein, called ZipA, serve as dual membrane tethers for FtsZ; without either one, FtsZ forms a ring but cannot divide, but if both FtsA and ZipA are missing, FtsZ remains diffuse in the cytoplasm ^3^. As FtsZ and FtsA are the most conserved across diverse bacterial species while ZipA is not, this review will focus on these two proteins.

## FtsZ, the tubulin homolog

The most highly conserved divisome protein is FtsZ, a tubulin homolog. FtsZ assembles into a polymeric ring-like structure at the inner surface of the cytoplasmic membrane, marking the future division site and organizing the assembly of the divisome complex ^4^. The ring then constricts in front of the invaginating division septum, reminiscent of the function of the actin ring in animal cells. Although early fluorescence microscopy studies suggested that FtsZ forms a continuous ring ^5^, more recent super-resolution microscopy demonstrated that the ring in *E. coli* actually consists of multiple patches several hundred nanometers apart in a circumferential path ^6, 7^. Other species as diverse as *Caulobacter crescentus*, *Bacillus subtilis*, *Streptococcus pneumoniae*, and *Procholorococcus* show similar patchy localization of FtsZ ^6– 10^, suggesting that a symmetrical, continuous ring of FtsZ is not required for cell division. Indeed, mutants of *E. coli* that constrain active FtsZ to a partial ring structure can still divide, although the cells display abnormal morphologies ^11, 12^. Recent studies have found that many bacterial species naturally initiate constriction on one side of the division site prior to becoming more symmetrical and that these asymmetric constrictions are likely promoted by relatively short FtsZ filaments that do not form a continuous ring structure ^13, 14^.

### Longitudinal interactions within FtsZ protofilaments

**Figure 1.**
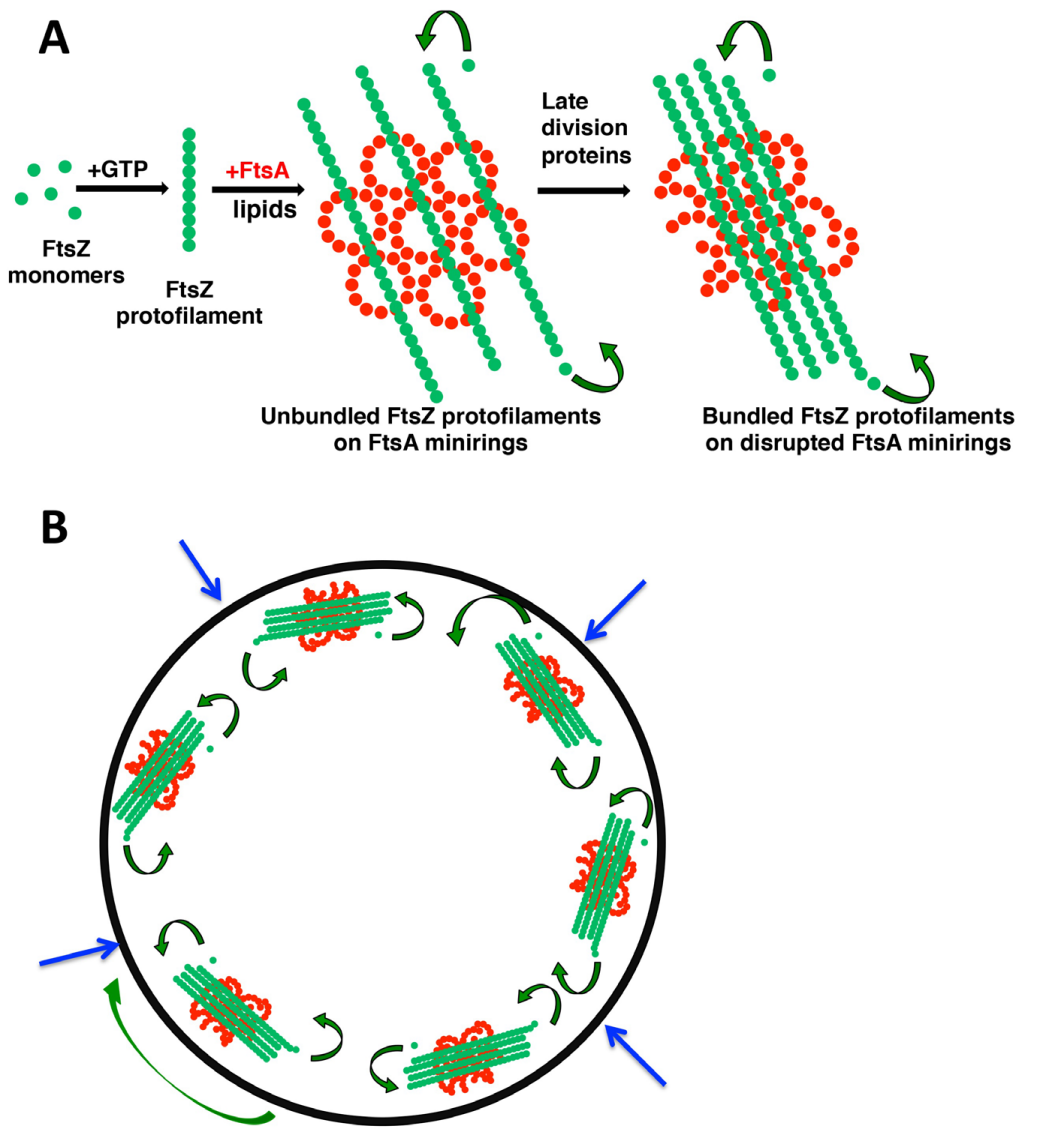
Model for how changing oligomeric structures of FtsA (red) and FtsZ (green) might regulate *Escherichia coli* cell division. A scheme is shown for how FtsZ and FtsA oligomeric states influence and potentially reinforce each other by positive feedback. Depicted are their structures on membranes and subunit exchange by treadmilling (curved arrows) ( **A**) and how several treadmilling complexes containing these structures at the inner surface of the cytoplasmic membrane might function in the cell for inward synthesis of the division septum ( **B**). The large green arrow denotes circumferential movement of a patch of FtsZ-FtsA; blue arrows show the direction of division septum synthesis that pushes the cytoplasmic membrane inward. FtsA, filamentous thermosensitive protein A; FtsZ, filamentous thermosensitive protein Z.

**Figure 2.**
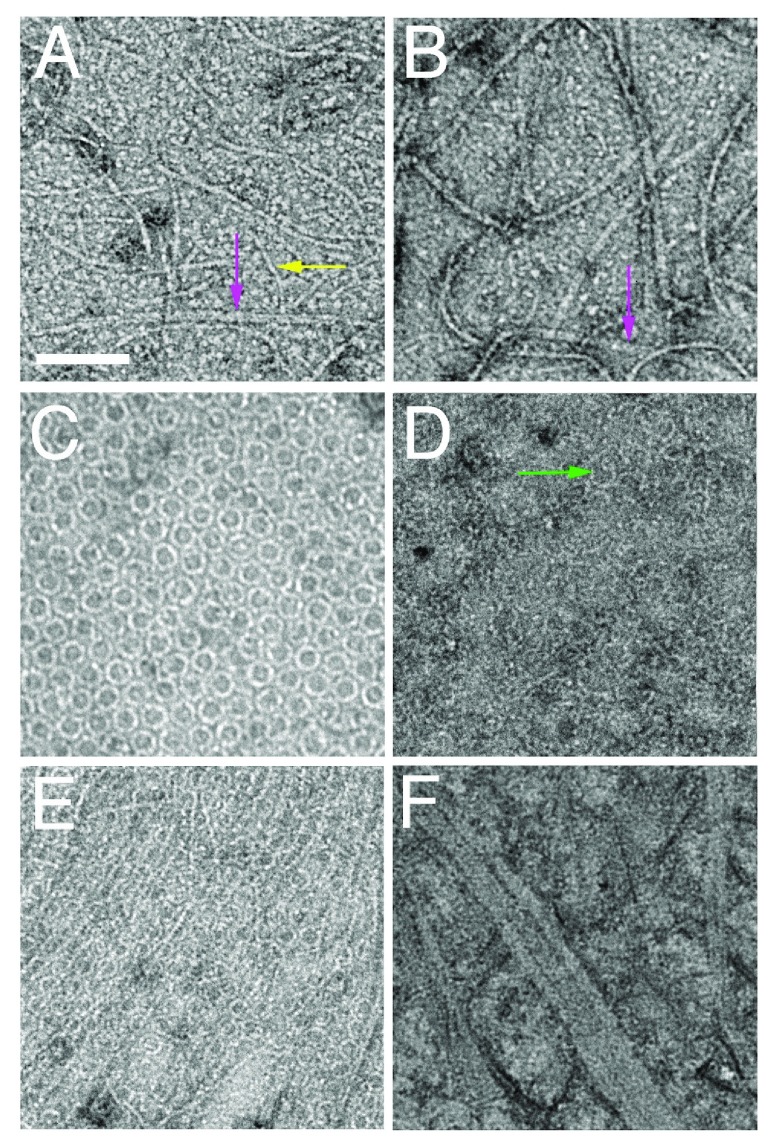
Oligomeric structures of FtsZ and FtsA. Shown are negatively stained transmission electron micrographs of FtsZ ( **A**) and FtsZ* ( **B**) from *Escherichia coli* and lipid monolayers containing minirings of *E. coli* FtsA ( **C**), FtsA* arcs ( **D**), FtsA + FtsZ ( **E**), and FtsA* + FtsZ* ( **F**). Scale bar = 100 nm. Arrows highlight double FtsZ protofilaments (magenta), single FtsZ protofilaments (yellow), or FtsA arcs (green). FtsA, filamentous thermosensitive protein A; FtsZ, filamentous thermosensitive protein Z.

Like tubulin, FtsZ binds and hydrolyzes GTP ^15, 16^ but forms long single-stranded protofilaments in the presence of GTP instead of hollow microtubules ^17^ ( Figure 1A and Figure 2A). Two recent reports demonstrated that in both *E. coli* and *B. subtilis* cells, FtsZ protofilaments exhibit polarity and undergo treadmilling ^18, 19^, during which subunits are selectively added to one end and removed from the other end ( Figure 1A). The result is that one or more FtsZ protofilaments migrate as a unit around the cell circumference, roughly following the path of the ring. Although the movement of each treadmilling filament is processive, other FtsZ protofilaments in the same cell can move in the opposing direction, so it seems that each protofilament unit has its own independent directionality.

FtsZ subunit turnover can occur within protofilaments or by treadmilling at protofilament ends. This turnover due to treadmilling is proportional to FtsZ’s GTPase activity, indicating that GTP hydrolysis regulates circumferential protofilament migration. Consistent with the role of GTP hydrolysis in protofilament turnover and disassembly ^20^, the thermosensitive mutant *ftsZ84* exhibits much lower GTP binding and hydrolysis activity than wild-type (WT) FtsZ and is unable to support cell division at 42°C ^15, 21^. Nevertheless, normal GTPase activity is clearly not essential for FtsZ function, as *ftsZ84* mutants form normal FtsZ rings and divide normally at the permissive temperature.

Through their interactions with other divisome proteins, FtsZ treadmilling protofilaments guide the key septal synthesis enzyme, FtsI (PBP3), which harbors the transpeptidase activity required for gradual inward cell wall growth eventually leading to cell splitting ^22, 23^. It is important to note that in *E. coli*, the activity of cell wall synthesis enzymes drives septum synthesis independently of FtsZ treadmilling but that *B. subtilis* septal wall synthesis is dependent on FtsZ treadmilling speed ^18, 19^. Although FtsZ subunits exchange rapidly within protofilaments as well as at the ends ^24, 25^, it remains to be seen exactly how internal subunits of FtsZ within a processively treadmilling filament help to guide a moving septum synthesis machine ^26^. One possible mechanism is that the septum synthesis machine continuously shuttles between different FtsZ subunits at the growing end, although it is difficult to imagine how this could occur processively. Recent evidence suggests that any physical links between FtsZ and the septum synthesis proteins are likely indirect ^27^.

It is also not clear how single-stranded FtsZ protofilaments treadmill (that is, although they have inherent polarity, why one end adds net subunits while the other loses them). A recent study suggests a possible mechanism. Examining crystal structures of FtsZ, Wagstaff *et al*. found a correlation between different conformational states of FtsZ and the ability to form polymers ^28^. The closed form of FtsZ has an incomplete subunit interface that correlates with the monomeric state, whereas the open form has a more complete subunit interface and can be modeled to fit into long straight protofilaments. This difference in interfaces between closed and open conformations of the subunits may result in distinct “on-off” rates from either end; if one end has a stronger “on” rate than the other, it would become the growing end of the filament. This interesting model awaits further experimental confirmation.

As FtsZ polymerization and treadmilling are essential for normal septal morphology, the inhibition of those activities should inhibit cell division. Indeed, there are numerous endogenous regulators that target FtsZ polymerization by various mechanisms, although inactivating any one of these regulators has only small effects on cell division. For example, the SOS-inducible SulA protein sequesters FtsZ subunits to prevent polymer formation or growth or both ^29– 31^. SlmA, a DNA-bound dimer that inhibits FtsZ polymerization over the nucleoid ^32, 33^, seems to function by binding to the conserved C-terminal peptide of FtsZ and subsequently severing the FtsZ filament ^34, 35^ similar to how another FtsZ inhibitor, MinC, depolymerizes FtsZ to prevent inappropriate division at cell poles ^36^. Interestingly, inactivating MinC in the GTPase-defective *ftsZ84* mutant significantly exacerbates its thermosensitivity ^37^. In addition, several peptides and small molecules, either natural or synthetic, target FtsZ polymerization and hold promise as potential antimicrobials ^38, 39^.

### Lateral interactions between protofilaments

In addition to longitudinal interactions between FtsZ subunits within a protofilament, lateral interactions between protofilaments play an important role. Such interactions, which result in crosslinking or bundling of FtsZ protofilaments, may be facilitated either by intrinsic attraction between FtsZ subunits or through accessory proteins. The general role of Zaps (Z-associated proteins), including ZapA, ZapC, and ZapD in *E. coli*, in promoting lateral interactions between FtsZ protofilaments is well described in recent reviews ^40, 41^ and will not be discussed in detail here. Although the loss of any one of these proteins has little effect on cell division, inactivation of more than one significantly reduces the division efficiency, suggesting that they have overlapping roles ^42^. However, it is not yet clear whether they work together or whether their mechanisms of action are distinct.

The physiological role of intrinsic lateral interactions between FtsZ protofilaments is less clear, although several interesting mutants of *E. coli* FtsZ provide clues. FtsZ _R85Q_, for instance, is partially defective for cell division and displays lower lateral interactions *in vitro* ^43, 44^. Another lateral mutant, FtsZ _R174D_, is non-functional for *E. coli* cell division and is unable to form bundled protofilaments *in vitro* when assembled in millimolar Ca ^++^, a condition that stimulates strong bundling ^45, 46^. Although this deficiency of FtsZ _R174D_ in bundling *in vitro* has been challenged recently ^47^, a genetic suppressor screen showed that this mutant protein can be rescued for function by amino acid changes at the nearby residue L169. Interestingly, protofilaments of FtsZ _L169R_, also called FtsZ*, make larger bundles than WT FtsZ *in vitro* ( Figure 2A, B) and exhibit lower GTPase activity ^48^. Consistently, FtsZ* was able to suppress the ability of *Kil*, a peptide from coliphage lambda that inhibits FtsZ function, to depolymerize FtsZ ^49, 50^. In addition, amino acid substitutions at residues E93 and D86 result in increased FtsZ filament bundling ^34, 51– 53^. Like the substitutions at L169, they are still able to function in cell division, although cells with these variants have defects, forming spiral-shaped FtsZ structures that cause abnormally shaped division septa and deformed cell poles. Hyper-bundled protofilaments have lower GTPase activities that should reduce treadmilling speed, thus perturbing the timing of septum synthesis relative to cell growth, leading to the observed shape abnormalities.

The accumulated data suggest a model in which lateral interactions between FtsZ protofilaments are regulated for optimal function. This is supported by the important role that intrinsic lateral interactions play in FtsZs from other species. For example, *B. subtilis* FtsZ protofilaments intrinsically bundle more than *E. coli* FtsZ, and these lateral interactions are mediated by the conserved C-terminal peptide of FtsZ ^54^. *C. crescentus* FtsZ also exhibits intrinsic lateral interactions mediated by its C-terminal domain ^55^. Moreover, *S. pneumoniae* FtsZ forms mostly double-stranded filaments, reflecting robust lateral interactions ^56^.

In addition to the Zap proteins, recent evidence indicates that other proteins specifically regulate lateral FtsZ interactions in diverse species. For example, ARC3, a protein in plant chloroplasts, antagonizes the filament bundling of FtsZ2, one of three FtsZ homologs used for chloroplast fission ^57^. FzlA and FzlC, both FtsZ-binding proteins in *C. crescentus*, regulate lateral interactions between FtsZ protofilaments ^55^, but the ZapA homolog in *C. crescentus* does not affect FtsZ lateral interactions *in vitro*, despite its ability to condense the FtsZ ring *in vivo* ^58^.

## FtsA, the actin homolog for cell division

FtsZ–FtsZ interactions, whether lateral or longitudinal, are clearly important for cell division. Nevertheless, several other proteins that interact directly with FtsZ also regulate FtsZ–FtsZ interactions. One is the aforementioned ZipA, which is narrowly conserved in the enterobacteriaceae and forms membrane-tethered homodimers ^59^. Another is SepF, which is present in a diverse group of Gram-positive bacteria. In *B. subtilis*, SepF assembles into sharply curved protofilaments at the membrane that regulate FtsZ assembly ^60, 61^ and has been proposed to act like a bacterial homolog of spectrin ^62^.

For the purposes of this brief review, we will focus on the most well characterized and highly conserved FtsZ interactor, FtsA, an actin homolog that binds ATP and forms polymers ^63– 66^. FtsA differs from actin in that it uses its C-terminal amphipathic helix for binding to the cytoplasmic membrane ^67^ and binds directly to the conserved C-terminal peptide of FtsZ ^68^. As a result, FtsA molecules provide important membrane tethers and potential guides for treadmilling FtsZ polymers. As FtsA binds to a region of FtsZ that also interacts with other key divisome proteins such as ZipA and is implicated in lateral interactions between protofilaments, it is important to understand how FtsA and FtsZ interact *in vitro*.

Until very recently, understanding this interaction was complicated by the difficulties in purifying active *E. coli* FtsA (EcFtsA) containing the amphipathic helix. However, earlier studies of FtsA from *S. pneumoniae* (SpFtsA), which was more amenable to purification, provided some important clues about FtsA function. Purified SpFtsA polymerizes bidirectionally in solution and its polymerization is facilitated upon membrane binding or when its C-terminal end is removed. Both polymerization and membrane attachment depend on ATP binding, suggesting that ATP induces a conformational switch to liberate FtsA’s C-terminal amphipathic helix for membrane binding and to induce polymerization ^69– 71^. Complementary physiological studies of SpFtsA are now catching up with its biochemical characterization ^72, 73^ which should greatly facilitate our understanding of FtsA function in this species and others.

Recent studies using purified EcFtsA (hereafter referred to as FtsA) indicate that it, too, can polymerize, but only on membranes and not as linear polymers ^74^. When added to lipid monolayers, FtsA assembles mainly into closed minirings containing 12 subunits each. These minirings, 20 nm in diameter, can be single or grouped into arrays ( Figure 2C). FtsZ, in the presence of GTP and near physiological concentrations, forms long single-stranded protofilaments that are strikingly aligned by these FtsA minirings but spaced apart by more than 15 nm ( Figure 2E). Notably, using fluorescence microscopy of purified FtsZ and FtsA on supported lipid bilayers, Loose and Mitchison ^75^ also observed the assembly of FtsZ into aligned polymer rafts on top of membrane-bound FtsA, although the ultrastructure of FtsA polymers was not examined. Consistent with the tendency of FtsZ protofilaments to curve under some conditions ^76– 78^, these aligned FtsZ polymers form large circular vortices that swirl unidirectionally by treadmilling. This behavior probably reflects the directional treadmilling of individual FtsZ complexes observed in cells ^18, 19^.

Just as mutants of FtsZ that decrease polymerization or GTPase activity are defective in cell division, mutants of FtsA that compromise the ATP-binding site or FtsA–FtsA interactions also have cell division phenotypes. Amino acid substitutions that inactivate EcFtsA function mostly map to the ATP-binding site and decrease ATPase activity, implicating nucleotide binding and hydrolysis in normal FtsA function ^79^. A very recent study showed that ATP binding and hydrolysis by *E. coli* FtsA promote membrane remodeling ^80^, analogous to the ability of ATP to promote membrane binding of *S. pneumoniae* FtsA ^71^. Such an activity might stimulate membrane invagination during septation. Likewise, residue substitutions at the subunit interface of *B. subtilis* FtsA that are predicted to affect FtsA oligomerization inhibit its cell division activity ^68^. Surprisingly, however, many substitutions at or near the subunit interface that decrease FtsA self-interaction actually enhance FtsA activity. For example, the R286W substitution in *E. coli* FtsA (FtsA*) results in a gain of function, bypassing the requirement for several other cell division proteins, including ZipA, and permits cells to divide at abnormally short cell lengths ^81– 83^. Other FtsA*-like substitutions have similar gain-of-function characteristics ^79, 84, 85^.

One model arising from these data proposes that, at least in *E. coli*, when an FtsA subunit is not engaged with another FtsA subunit in an oligomer, it is free to interact with later-division proteins, helping to drive cytokinesis forward ^84^. In support of this idea, purified FtsA* does not form closed minirings on lipid monolayers but instead mostly short arcs with the same curvature ( Figure 1A and Figure 2D) ^74^. These arcs, with potentially free protein domains available at subunit ends unlike in minirings, might be more likely to interact with late-division proteins ^84^. In addition, the dynamics of treadmilling might be significantly influenced by the oligomerization state of FtsA on the membrane and its ability to tether FtsZ to the membrane (see “A role for FtsA in regulating lateral interactions between FtsZ protofilaments” section below). Future high-resolution electron microscopic studies with purified FtsA bound to other cell division proteins on membranes should help to test this model and to elucidate factors that regulate treadmilling dynamics.

## A role for FtsA in regulating lateral interactions between FtsZ protofilaments

As mentioned above, minirings of *E. coli* FtsA inhibit lateral interactions between FtsZ protofilaments. If FtsZ bundling is important for cell division to proceed, the prediction is that FtsA minirings switch to a form that promotes FtsZ bundling. FtsA* may be an example of this form because when FtsZ is assembled on lipid monolayers seeded with FtsA* arcs, FtsZ protofilaments are no longer separated by more than 15 nm and instead become highly bundled, with lateral distances of about 7 nm ^74^. This stimulation of FtsZ bundling by FtsA* has also been observed in solution ^86^, indicating that some behaviors in solution may mimic effects seen on the membrane. Strikingly, when the FtsZ* hyper-bundled variant is assembled on lipid monolayers with FtsA*, large sheets of FtsZ* with protofilaments spaced about 5 nm apart are formed ( Figure 2F). This over-bundling suggests that FtsA*-mediated bundling of FtsZ is synergistic with the intrinsically increased bundling of FtsZ*. The physiological consequences are clear, as cells with both FtsA* and FtsZ* do not divide normally, often exhibiting twisted cell division septa ^48^. Interestingly, when FtsZ* is added to lipid monolayers containing FtsA minirings, the minirings become disrupted ^74^, hinting at a positive feedback loop (see further below in this section).

It is not yet known which molecular mechanisms control either intrinsic or FtsA-mediated FtsZ lateral interactions or which residues are involved in direct contacts. Nonetheless, such interactions likely involve not only the core polymerizing domain and C-terminal peptides of FtsZ but also the conserved, intrinsically disordered linker that connects them ^87– 90^. Recent results indicate that changing the length of the linker in *E. coli* FtsZ can also alter the distance between bundled protofilaments, although the effects on lipid membranes with natural FtsZ tethers have not yet been explored ^90^.

An attractive model to explain these results is that FtsA minirings antagonize bundling of FtsZ protofilaments by holding them apart for some period of time and that this constraint is a key checkpoint in the progression of cell division in *E. coli* ( Figure 1A). In this model, gain-of-function variants of FtsA such as FtsA* ignore this checkpoint because they do not form minirings on the membrane and instead would transition directly to FtsA*-like non-miniring oligomers. One consequence of this transition would be premature bundling of FtsZ protofilaments. Although the overall morphology of the FtsZ ring does not change much during constriction ^8, 9, 91^, a more condensed state of FtsZ ^91, 92^, potentially caused by increased protofilament bundling, is thought to be important for promoting septum formation ( Figure 1B). Indeed, in FtsA*-like mutants that may prematurely bundle FtsZ, cell division is accelerated, potentially by bypassing a checkpoint, resulting in cells that divide at shorter cell lengths and overcome perturbations to the normal cell division pathway such as inactivation of ZipA. Considering the ability of bundled FtsZ to disrupt FtsA minirings *in vitro* ^74^, we propose that factors that bundle FtsZ and factors that disrupt FtsA minirings act in the same pathway to promote septation. As FtsZ becomes more bundled, FtsA minirings are disrupted, which promotes more FtsZ bundles, which in turn disrupts more FtsA minirings. Therefore, according to this model, the increased bundling of FtsZ and decreased oligomerization of FtsA reinforce each other in a positive feedback loop that helps to maintain constriction of the inner membrane in the forward direction in concert with septum synthesis. Genetic evidence suggests that FtsEX, an ATPase complex that senses septum synthesis but also contacts FtsZ and FtsA, may be involved in such a feedback loop, as the FtsX protein likely regulates FtsA’s oligomerization state ^93, 94^. As more mechanistic details in the FtsA–FtsZ interaction are revealed, interesting parallels with actin and tubulin in eukaryotic cells should emerge. For example, FtsA control of FtsZ bundling is reminiscent of F-actin bundling by septins ^95^.

Although the above model is based on *in vitro* data and FtsA minirings have not yet been observed in *E. coli* cells, other genetic data support the idea that FtsA antagonizes FtsZ bundling. For example, a moderate excess of FtsA in *E. coli* cells lacking the FtsZ bundling proteins ZapA or ZapC significantly exacerbates the cell division defects caused by ZapA/C deficiency alone; conversely, excess FtsA can rescue cell division deficiencies in cells with over-bundled FtsZ caused by excess ZapA ^74^. FtsA minirings are reminiscent of the structures formed by *B. subtilis* SepF but differ from the straight filaments formed by *T. maritima* FtsA reported by Szwedziak *et al*. ^96^. We speculate that FtsA proteins from distinct bacterial species assemble into a variety of forms and that the miniring form is potentially exclusive to *E. coli* and related gamma-proteobacteria that have multiple FtsZ membrane tethers but need to impose a checkpoint delay on septum formation. In bacteria with only one membrane anchor, perhaps FtsA polymerization into straight actin-like filaments serves to tether more FtsZ to the membrane and consequently reduce its treadmilling, keeping septation in check by a different mechanism.

Interestingly, gain-of-function mutants in other essential *E. coli* divisome genes ( *ftsL* and *ftsB*) have similar properties as ftsA*, hinting that other parts of the cell division apparatus, including multiple periplasmic proteins with no known enzymatic activities, are also involved in regulating the progression of septum formation ^85, 97^. It is notable that the FtsZ bundling-stimulator FtsA* can rescue the toxicity caused by excess ZapC, which is the opposite of what would be predicted if ZapC also induces FtsZ over-bundling ^98^. These effects may reflect the increased ability of FtsA* to recruit downstream divisome proteins. Clearly, the effects of gain-of-function mutants such as FtsA* cannot be limited to FtsZ bundling alone.

## Conclusions and perspectives

We have discussed important recent breakthroughs in our understanding of how FtsZ and FtsA interact to organize bacterial cell division, mainly in *E. coli* but also increasingly in other model systems. FtsZ protofilaments treadmill around the circumference of the division ring and are connected—directly or indirectly—to the membrane and periplasmic peptidoglycan synthesis machinery by FtsA and other proteins to construct the septum. The next key challenge is to elucidate the mechanism of treadmilling and how it guides septum synthesis. In addition, important new evidence for the role of lateral interactions in FtsZ function and the ability of FtsA’s oligomeric state to regulate these interactions, including the roles of ATP and GTP in these processes, needs to be explored more in detail. These experiments should be greatly facilitated by improved lipid membrane systems, such as lipid monolayers, supported lipid bilayers, and lipid discs, as well as rapidly improving direct visualization methods such as super-resolution microscopy and cryo-electron tomography. Finally, it is not yet settled whether the primary purpose of dynamic FtsZ filaments is to generate a physiologically relevant inward constriction force, supported by experiments with liposomes ^99^, or to interface with the septal synthesis machinery and respond flexibly to numerous inputs ^100, 101^ or to do both.

## Abbreviations

ATP, adenosine 5′ triphosphate; EcFtsA, *Escherichia coli* FtsA; FtsA, filamentous thermosensitive protein A; FtsZ, filamentous thermosensitive protein Z; FzlA, FtsZ-localized protein A; FzlC, FtsZ-localized protein C; GTP, guanosine 5′ triphosphate; SepF, septation protein F; SlmA, synthetic lethal with Min protein A; SpFtsA, *Streptococcus pneumoniae* FtsA; WT, wild-type; ZapA, ZapC, ZapD, FtsZ-associated proteins A, C, D; ZipA, FtsZ-interacting protein A.
